# Optimizing peer-led health interventions: A social network analysis approach for identifying influential fishermen in Malawi

**DOI:** 10.1371/journal.pgph.0007185

**Published:** 2026-09-09

**Authors:** Madalo Mukoka, Alison Price, Marriott Nliwasa, Owen Mhango, Mphatso Mwapasa, Moses K. Kumwenda, Hussein H. Twabi, Robina Semphere, Takondwa C. Msosa, Chisomo Msefula, Augustine T. Choko, Guy Harling, Katherine Fielding

**Affiliations:** 1 Helse Nord Tuberculosis Initiative, Department of Pathology, Kamuzu University of Health Sciences, Blantyre, Malawi; 2 Department of Infectious Disease Epidemiology and International Health, London School of Hygiene and Tropical Medicine, London, United Kingdom; 3 Malawi-Liverpool-Welcome Clinical Research Programme, Blantyre, Malawi; 4 Malawi Epidemiology and Intervention Research Unit, Lilongwe, Malawi; 5 Department of Population Health, London School of Hygiene and Tropical Medicine, London, United Kingdom; 6 School of Public Health, University of the Witwatersrand, Johannesburg, South Africa; 7 Institute of Resilient Health Systems, Department of International Public Health, Liverpool School of Tropical Medicine, Liverpool, United Kingdom; 8 Institute of Life Course and Medical Sciences, University of Liverpool, Liverpool, United Kingdom; 9 School of Health and Wellbeing, University of Glasgow, Scotland, United Kingdom; 10 Department of Global Health, Amsterdam University Medical Centre, Amsterdam, The Netherlands; 11 Department of International Public Health, Liverpool School of Tropical Medicine, Liverpool, United Kingdom; 12 Institute for Global Health, University College London, London, United Kingdom; 13 Africa Health Research Institute, KwaZulu-Natal, South Africa; 14 MRC/Wits Rural Public Health & Health Transitions Research Unit (Agincourt), University of the Witwatersrand, Johannesburg, South Africa; 15 School of Nursing & Public Health, University of KwaZulu-Natal, KwaZulu-Natal, South Africa; PLOS: Public Library of Science, UNITED STATES OF AMERICA

## Abstract

Social network interventions (SNIs) can leverage influential individuals within personal networks to amplify health behavior adoption and intervention diffusion. While SNIs are potentially effective in enhancing uptake of HIV prevention and other interventions, identifying optimal peer leaders remains challenging. This study assessed how social network-based approaches can improve peer leader identification compared to non-structural approaches (i.e., those that do not use network data for promoter selection). Between 8^th^ October 2024 and 31^st^ March 2025, we mapped the social connections among fishermen in two communities in Mangochi, Malawi. The communities had previously participated in a cluster-randomized trial (FISH), which aimed to create demand among fishermen for HIV and schistosomiasis services via peer-nominated leaders. Using Network Canvas and a photographic census, we conducted a network survey, capturing ties, support roles and interaction frequency. Whole-network maps were constructed, centrality measures and the key player problem positive (KPP-POS) algorithm were applied to identify the highest-ranking individuals as potential promoters. We compared the reach (i.e., proportion of nodes within two steps of a promoter) of peer-nominated leaders selected by FISH to these sociometric methods**.** We recruited 370 of 397 eligible fishermen (mean age 34.8years [SD 13.11]). Both communities exhibited sparse, low-reciprocity networks with long path lengths. One network had two dense cores, while the other featured a single core. There was no evidence of assortative mixing by education, age or village of residence. FISH trial peer leaders were not consistently central in the constructed maps. The KPP-POS algorithm identified alternative, more dispersed nodes, achieving the highest reach (66% in F001; 75% in F024) versus in-degree (58%, 67%), closeness (58%, 68%), betweenness (58%, 69%) and eigenvector (60%, 68%) promoter sets. Our findings highlight that strategically identified promoters can achieve good reach within social networks, crucial for effective public health programming in complex settings like fishing communities.

## Introduction

Social network interventions (SNI) leverage social networks or social network data by using social influence to accelerate the adoption of ideas, behaviors or information among interconnected individuals [1]. Network interventions are relevant in contexts where health systems often face constraints in delivering targeted interventions, and access to formal health information is limited [2,3]. In these contexts, they facilitate peer-to-peer knowledge exchange, promote uptake of health services, and support behavior change. To maximize their impact, these interventions target individuals who, by virtue of their position or role in the network, are considered influential [1,4,5]. These influential individuals are, in turn, trained to promote the intervention of interest in the network.

Fishing communities in sub-Saharan Africa bear a disproportionate burden of HIV and schistosomiasis [6,7]. Fishermen often experience higher HIV prevalence than the general population, due to high mobility, limited access to health services, transactional sex, and occupational risk environments [7–9]. Frequent contact with infested freshwater during fishing also places fishermen at increased risk of schistosomiasis infection [10]. Despite the availability of effective public health services for both conditions, uptake remains suboptimal [10]. Given the strong social ties and interconnected nature of fishing communities, SNIs offer a promising approach for promoting health information, increasing service uptake, and supporting behavior change among fishermen [11,12].

The effectiveness of network interventions is grounded in multiple theoretical frameworks, including the diffusion of innovation theory, which suggests that individuals are more likely to adopt a new idea or behavior if one or more individuals in their close social network has already done so [13]. Evidence from multiple studies supports the role of social networks in influencing behavior change and the uptake of health interventions. For example, social influence within networks in various studies led to a five-fold increase in contraceptive uptake, led the spread of obesity in a large cohort, led to reduction in smoking among adolescents, increased ART initiation, and improved HIV testing uptake among female sex workers [2,14–17]. These findings cement the significance of social networks in influencing behavior change, and further, uptake of novel interventions.

Identifying individuals who can efficiently promote an intervention in a network is crucial in optimizing a SNI [18]. However, it is not always clear who in the network possesses the right qualities to influence the uptake of interventions. Strategies to identify such individuals range from peer-nomination, external identification of opinion leaders, and positional approaches - all of which implicitly rely on network information - to approaches that explicitly use individuals’ network attributes following network analysis [1,19–21]. Using social network analysis (SNA) to identify optimal individuals requires the collection of data on interpersonal ties, mapping the ties and calculation of network measures [1,22,23]. Various network measures exist but the most commonly used is high in-degree centrality (number of times a person is nominated by others in the network), on the basis that highly nominated individuals are popular and therefore can create cascades necessary to spread the intervention in a network [3,24–26]. This and other related approaches have been successfully applied in HIV prevention and other public health programs [15,27,28]. For example, one study identified peer leaders using social network analysis following a whole network survey and found that interventions delivered by the selected peer leaders improved the uptake of HIV testing by 35% compared to control groups [29].

Although SNIs have been widely implemented in HIV prevention and other public health programs in low- and middle-income countries (LMICs), including among fishing communities, the methods used to identify peer leaders vary considerably [30]. In many studies, peer leaders are selected through peer nomination, staff recommendations, or self-selection, often without a systematic assessment of their position within the social network structure [17, 19, 31, 32]. While these approaches can be effective, there is limited evidence on whether network-informed selection strategies can identify individuals with greater potential to reach and influence others, particularly in highly mobile populations such as fishermen. Furthermore, few studies in LMIC settings have directly compared different network-based approaches for identifying influential individuals within complete sociocentric networks [33]. Addressing these gaps is important for optimizing the design of future network-based interventions. Thus, this study aimed to assess how network analysis can be used to pinpoint optimal promoters for future health interventions among fishermen by mapping social connections. We compared how SNA-informed promoter identification performed against non-structural approaches (i.e., those that do not use network data to inform promoter selection), thus contributing to the growing body of evidence on network-based public health strategies and providing practical insights for implementing network-based interventions in high-risk, mobile populations.

## Methods

### Setting

The study was conducted in Mangochi District, located in the southern tip of Lake Malawi, with fishing activities concentrated along the lakeshore [34]. Recruitment of participants was limited to fishermen and conducted at two clusters (also known as landing sites: parts of land from which fishermen dock) that were previously used within the FISH cluster-randomized trial (Creating demand for Fishermen’s Schistosomiasis and HIV services). The FISH trial (trial registration: ISRCTN14354324) was conducted between March 2020 and January 2023 and compared three strategies for creating demand for HIV and Schistosomiasis services: peer educators (PE); peer-distributor-educators (PDE); and beach-side services alone (standard of care). Two of the strategies, PE and PDE, used peer-nomination of influential fishermen to deliver information on HIV and schistosomiasis, distribute invitation coupons, and provide HIV self-testing kits (PDE arm alone) to fellow fishermen in their boats [35]. The FISH trial included 45 clusters, each comprising multiple boat crews (10 crews per cluster), each with multiple members (approximately 10 members per boat crew).

This study focused on clusters that recruited participants into the intervention arms (PE or PDE arms) of the FISH trial. Two clusters were purposively selected from 30 (i.e., 15 clusters from PE arm and 15 from PDE arm), based on trial outcome HIV testing uptake (best and worst performing). We selected these clusters to explore whether differences in network structure and the characteristics of influential individuals could help explain these contrasting outcomes. We thus included clusters F001(PDE) and F024 (PE), representing the best and worst performing clusters, respectively.

### Data collection

Due to non-existence of current registries for active fishermen, we enumerated all consenting, permanently resident fishermen (i.e., those who had resided in the area for the past 12 months and planned to stay for 6 more months) aged 18 years and above. We had planned to only include fishermen who had participated in the FISH trial, but the dynamic nature of the population meant this would have given an incomplete current network.

Data was collected between 8^th^ October 2024 and 31^st^ March 2025. During the enumeration exercise, potential participants were identified with the support of FISH trial peer leaders. Recruitment occurred over a period of one month at the lakeshore primarily during the evenings before fishermen departed for fishing, in the mornings upon their return and occasionally in the surrounding villages. Participants were asked to provide personal identifying information, such as names, nicknames, age, village of residence and level of education. Participants were also asked about their HIV and schistosomiasis history and circumcision status. This information was used to prepare cluster-specific name rosters. Additionally, we prepared a photographic census of all consenting participants to facilitate data collection during the subsequent network survey, as a prior pilot study had shown that sometimes participants shared names and, in some cases, did not accurately know the personal identifying information of their close contacts. The name roster and photographic census data were held separately but linked through participant identification numbers (PIDs).

One month after the enumeration and photographic census, we contacted all consenting participants to conduct the survey. We administered the survey to all fishermen who were part of our rosters using the open-source Network Canvas app [36]. The survey was administered face-to-face by trained research assistants, in Chichewa (a local language) using a tool that had been back-translated and pilot-tested in another cluster that was not included for participation in this study. First, respondents were asked to name as many social contacts (referred to as “alters”) as desired among the listed fishermen in their cluster who they considered to be closest to them or were otherwise important to them. The participants were provided with photographic census data to see who was listed in their cluster, with the images labelled with study-assigned PIDs. Participants could either provide a name or nickname of their close contact or PID from the photographic census data. The details were used to conduct a logic search of the name roster held on an encrypted tablet and return a shortlist of potential matches. Once the alter was identified, name interpreter questions were asked to capture characteristics such as relationship with the respondent, frequency of interaction, and whether they had ever discussed or received advice on HIV or schistosomiasis. Participants also indicated the specific types of support provided by each alter across five key domains: health advice (whom you turn to for advice including health decisions); emotional (whom you rely on for comfort); financial (whom you rely on to help with money); confides in (whom you talk to about private matters); and social (whom you enjoy socializing with) [37].

### Network measures

Data were analyzed, separately by cluster, using R statistical software [38]. In each cluster, sociocentric networks were constructed by matching contacts named by each participant to other enlisted cluster members. PIDs were used to match the nodes (individual actors in the network), facilitating the construction of a complete sociocentric network map. A tie (or edge) represented the relationship between two nodes in the network, while a triad referred to the relationship involving three nodes.

In each cluster, seven network measures were calculated to assess network cohesion, diffusion potential, and overall connectedness to inform optimal promoter identification: multiplexity, egonet density, assortativity coefficient, size, overall density, average path length, transitivity, and reciprocity [3,39].

At the tie level, multiplexity was calculated as the cumulative number of support domains (health advice, emotional support, financial assistance, confidential sharing, and social companionship) provided within each connection. At the node level, the assortativity coefficient was calculated as the correlation between the attributes (age, village of residence, education level) of connected nodes, measuring the tendency of nodes to connect with others sharing similar attributes (range: -1 for perfect disassortative mixing to 1 for perfect assortative mixing) while egonet density was calculated as the proportion of actual ties among an ego’s alters relative to the total number of possible ties between them (range: 0 where none of the alters are connected to 1 where all alters are connected). At whole network level, network size was measured as the total number of nodes and ties reported; overall density was measured as the ratio of observed ties to the total possible ties in the network (range: 0 for fully disconnected network to 1 fully connected network); The average path length was the average of the distances between all the nodes in the network; transitivity was calculated as the proportion of closed triads relative to the total number of possible triads in the network (range: 0 for no triads to 1 where every connected triple forms a triangle) and reciprocity was calculated as the proportion of bidirectional ties among all ties in the network (range: 0 for purely unidirected map to 1 where all edges have a corresponding mutual edge).

### Data analysis

To identify alternative potential groups of peer promoters within each network, we computed four centrality measures—degree, closeness, betweenness, and eigenvector centrality—and selected the top 5% highest-ranking nodes (referred to as the promoter set) for each measure [3,33,40–42]. In-degree measures the number of nominations a node receives from other nodes; closeness measures the average distance a node is from all other nodes in the network indicating their potential to reach others through relatively few steps; betweenness measures the degree a node lies on the shortest paths connecting other nodes and reflects their potential to bridge otherwise disconnected groups within the network, and eigenvector measures of a node’s influence based on the centrality of its connected neighbors, identifying individuals connected to other well-connected network members [3,43]. As an additional selection method, we applied the Key Player Problem Positive (KPP-POS) algorithm to identify the top 5% optimal nodes [22]. This method, unlike traditional centrality measures that evaluate nodes individually, considers the combined influence of a group of nodes, optimizing selection to maximize overall network coverage and cohesion while avoiding structurally equivalent or redundant nodes [22].

For each promoter set, we compared their demographic characteristics to those of the general population. Additionally, within each cluster, we quantified the similarity between promoter sets by calculating the Jaccard similarity score, which measures the extent to which different promoter identification approaches selected the same fishermen. The score is calculated as the proportion of promoters shared between two sets relative to the total number of unique promoters identified across both sets (i.e., the ratio of the size of their intersection to the size of their union), with higher scores indicating greater agreement in the individuals selected [44]. For each map, we identified the FISH trial peer leaders. For the peer leaders and all five other potential promoter sets, we calculated the distance on the network map from each promoter to all other nodes and highlighted the nodes that were within two steps. Finally, we compared the reach (i.e., the proportion of nodes within two steps of the promoters) of the FISH peer leaders and the promoters selected following network analysis. Analysis and visualization were done using the igraph package in R (version 4.4.1) [38]

### Ethics

The study was approved by Kamuzu University of Health Sciences Research and Ethics Committee, approval number: P.05/24–0799 and London School of Hygiene & Tropical Medicine Ethics committee, approval number: 31119. All participants gave written informed consent in the local language or a witnessed consent plus thumb-print if illiterate before participation. An impartial witness, identified by illiterate participants, read and explained the study information before consent was given.

### Inclusivity in global research

Additional information regarding the ethical, cultural, and scientific considerations specific to inclusivity in global research is included in the Supporting Information (S1 Checklist).

## Results

**Fig 1:**
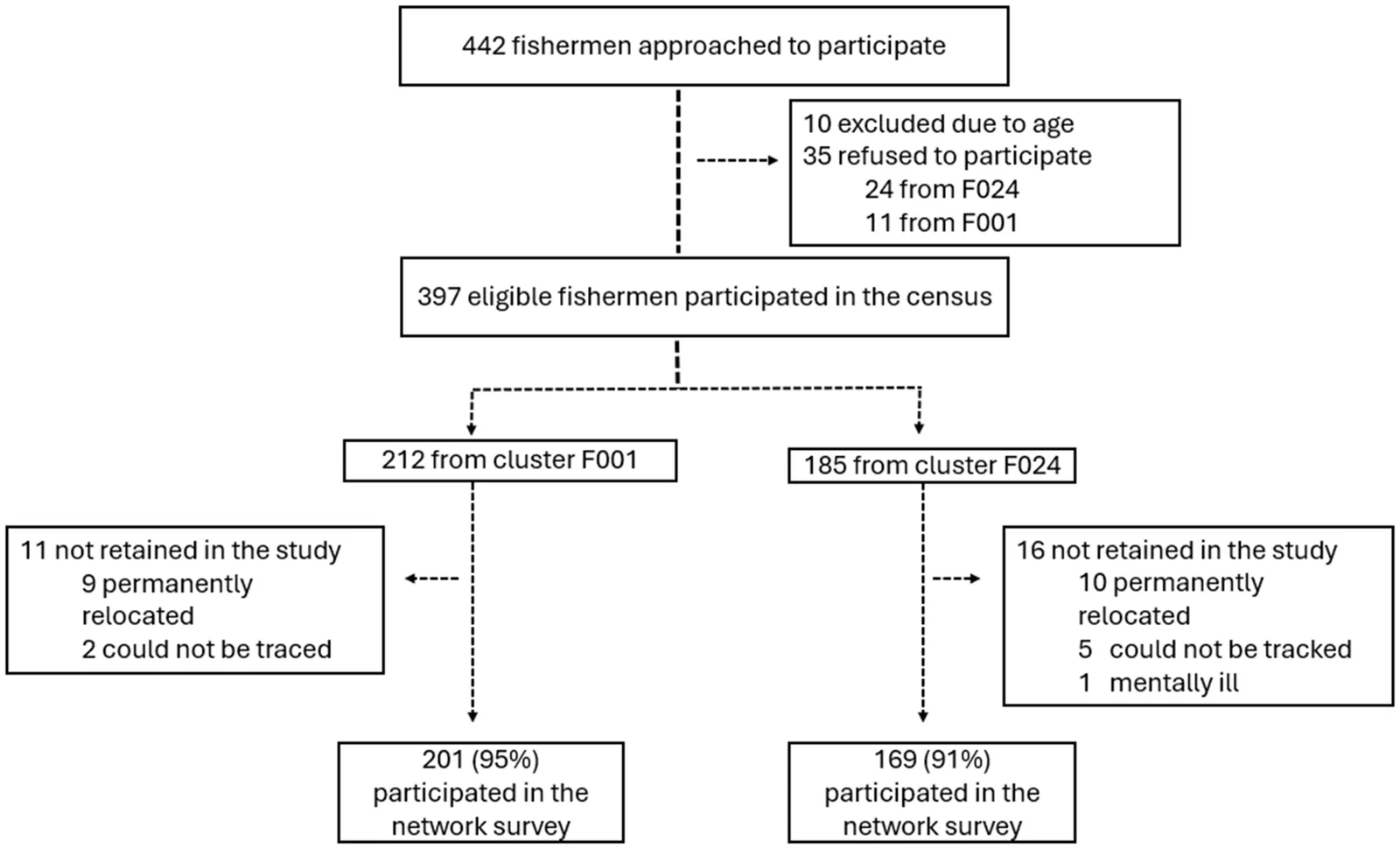
Study profile.

We approached a total of 442 fishermen (226 from cluster F001 and 216 from F024) and enumerated a total of 397 fishermen (212 in F001 [94%] and 185 in F024 [86%]) (Fig 1). Of the 397 census participants, we successfully re-contacted and consented 370 individuals one month later to participate in the network survey.

Participants from cluster F001 were on average older, more literate and a higher percentage had ever tested for HIV and in the past year compared to those in F024 (Table 1). Nearly all who self-reported a past positive HIV test were on Antiretroviral Therapy (ART). More participants had received any form of circumcision in F024 (74.6%) than in F001 (48.8%). Almost all the participants had experienced symptoms of schistosomiasis, but traditional treatment was used more often in F024 (68.0%) than in F001 (47.8%).

**Table 1:** Sociodemographic characteristics of participants by study cluster.

	F001	F024
n	201	169
Age mean (sd)	37.4 (14.7)	31.7 (10.0)
Able to read one page?		
Yes	183 (91.0)	144 (85.2)
Marital status (n [%])		
Married	115 (57.2)	89 (52.7)
Living as married	6 (3.0)	6 (3.6)
Never married	53 (26.4)	45 (26.6)
Divorced or separated	23 (11.4)	28 (16.6)
Widowed	4 (2.0)	1 (0.6)
Highest education level attained (n [%])		
None	10 (5.0)	9 (5.3)
Primary	86 (42.8)	110 (65.1)
Secondary	101 (50.2)	49 (29.0)
Tertiary	4 (2.0)	1 (0.6)
Ever tested for HIV?		
Yes	191 (95.0)	146 (86.4)
Tested in the last 12 months?^†^		
Yes	122 (63.9)	84 (57.5)
Self-reported HIV result (n [%])		
Positive	28 (14.7)	29 (19.9)
Negative	163 (85.3)	116 (79.5)
Doesn’t remember	0	1 (0.7)
ART treatment^‡^		
Yes	28 (100)	28 (96.6)
No	0	1 (3.4)
Circumcision		
Voluntary Male Medical Circumcision	74 (36.9)	34 (20.2)
Traditional	24 (11.9)	92 (54.4)
None	103 (51.2)	43 (25.4)
Ever had schistosomiasis symptoms?		
Yes	192 (95.5)	162 (95.9)
Ever taken traditional treatment for schistosomiasis?		
Yes	96 (47.8)	115 (68.0)
No	101 (50.2)	51 (30.2)
Not sure	4 (2.0)	3 (1.8)

Data in parentheses are column percentages. ^†^Asked of those who had ever tested ^‡^ Asked of those who self-reported an HIV reactive status

sd standard deviation, ART antiretroviral therapy

**Fig 2:**
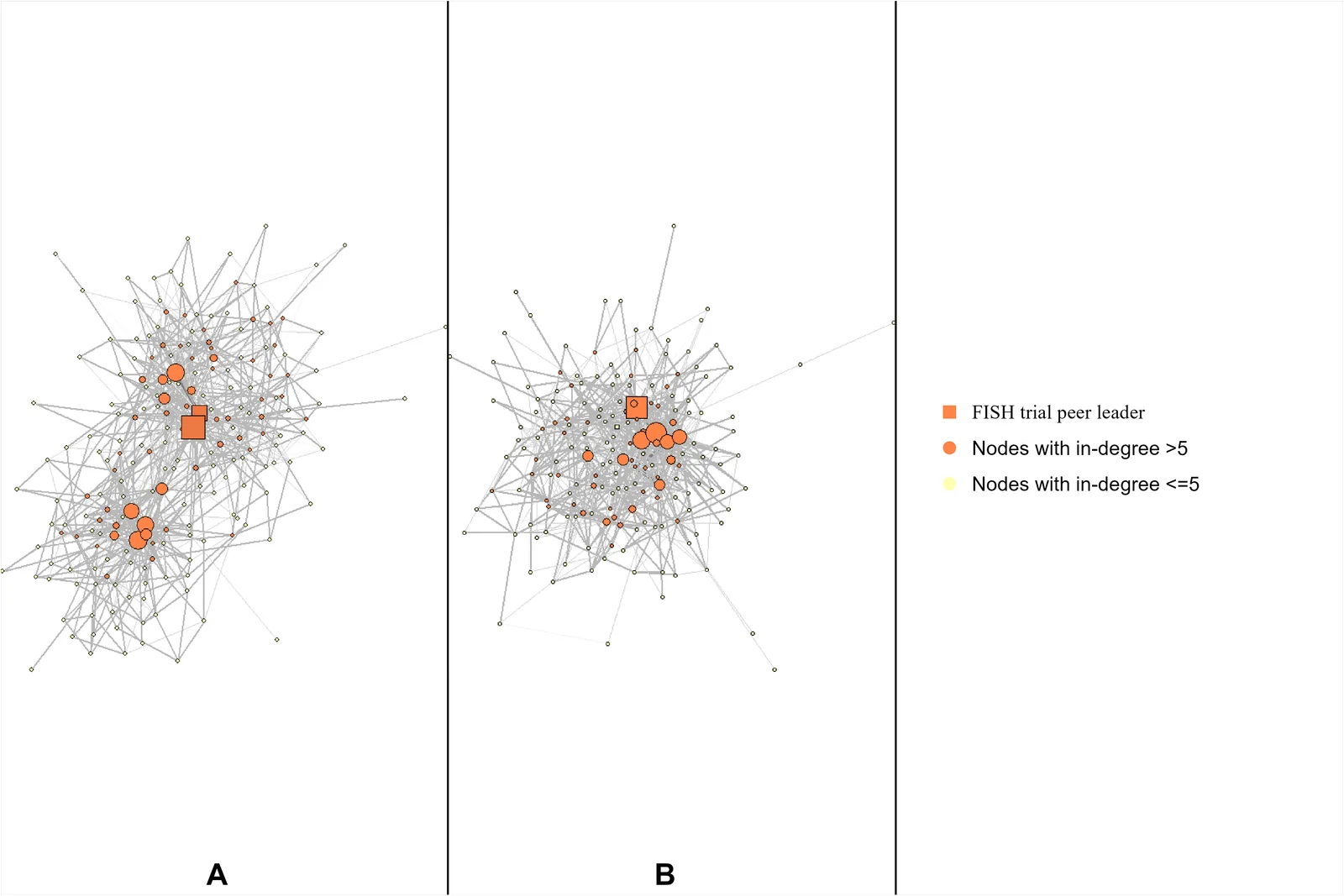
Social networks of fishermen within geographically distinct study clusters in Mangochi, Malawi. Each circle represents a fisherman (node), and the size of the circle is proportional to in-degree centrality (the number of nominations received). Line thickness is proportional to multiplexity.

The social network of cluster F001 was larger, with 201 nodes and 911 ties, while F024 had 169 nodes and 792 ties (Table 2). F001’s network was broadly cohesive but had two dense cores connected by numerous overlapping edges (Fig 2A), while F024’s featured a large single core with a small periphery (Fig 2B). Notably, the peer leaders from the FISH trial in F001 were positioned on one side of the network core. The networks were structurally similar, with long path lengths, sparsely connected globally but locally clustered (Table 2). There was no assortative mixing based on education level, age and village of residence in either network and in both clusters, over half of the ties involved all five support domains (Table 3).

**Table 2:** Node level and whole network descriptive data, by study cluster.

	Cluster name	
**Measure**	**F001**	**F024**
Size	Node count = 201 Tie count = 911	Node count = 169 Tie count = 792
Overall density^†^	0.022	0.025
Egonet density^†^	0.240	0.247
Average path length	18.96	18.91
Transitivity^†^	0.156	0.165
Reciprocity^†^	0.058	0.063
Assortativity coefficient^‡^		
Village of residence	-0.026	-0.007
Age	0.006	0.00004
Education	0.027	0.019

^†^possible range 0–1; ^‡^ possible range -1 to +1,

**Table 3:** Multiplexity measures by cluster.

Multiplexity*	F001	F024
1	18%	18%
2	3%	3%
3	12%	13%
4	11%	15%
5	56%	51%

* *Multiplexity values are the cumulative number of support domains (health advice, emotional support, financial assistance, confidential sharing, and social companionship)*

### Promoter Identification

This process involved selecting key nodes within the two clusters, using multiple measures: in-degree, closeness, betweenness, eigenvector, and KPP-POS. For each measure, the top 5% nodes were identified as potential promoters based on their scores (S1 Fig). In cluster F001, 24 unique nodes were selected across all five measures (S2 Fig). Eight nodes appeared across three or more measures while only two nodes appeared across all five measures (S2 Fig). In cluster F024, a total of 30 nodes were selected across the five measures. Six nodes appeared across three or more measures with only two nodes appearing across all measures (S3 Fig).

Comparing FISH trial peer leaders to sociocentric selections, we found that in F001, one of the peer leaders was selected by four of the five sociocentric measures, while the other was selected by three measures. In F024, one peer leader was chosen by all of the sociocentric measures, however the other was selected by none (S1 Fig).

### Demographic characteristics of promoters

In both clusters, selected nodes were on average five years older than the study population except for the KPP-POS set whose mean age was lower (S1 Table). Literacy among promoters was comparable to the study population. HIV testing rates were higher across all promoter sets compared to the study population (S1 Table).

### Relationship between selected promoter sets

Jaccard similarity scores quantified the overlap in individuals identified as promoters across different promoter selection approaches, with higher scores indicating greater agreement in the individuals selected. Overall, similarity scores ranged from 0.11 to 0.54, with betweenness consistently having low similarity scores with other measures. Other pairs varied between 0.25 and 0.54 (Table 4). In cluster F001, in-degree and eigenvector centralities showed the highest similarity (Jaccard score = 0.54). KPP-POS had moderate similarity with in-degree (0.33), closeness (0.33), and eigenvector (0.25). In cluster F024, in-degree and closeness had the highest similarity (0.54). KPP-POS showed moderate overlap with in-degree (0.33) and closeness (0.25), but low similarity with betweenness (0.11) and eigenvector (0.18) –Table 4

**Table 4:** Similarity scores between selected nodes, by study cluster.

	F001				F024			
	Closeness	Betweenness	Eigenvector	KPP-POS	Closeness	Betweenness	Eigenvector	KPP-POS
**In-degree**	0.43	0.43	0.54	0.33	0.54	0.11	0.43	0.33
**Closeness**	–	0.33	0.25	0.33	–	0.11	0.33	0.25
**Betweenness**	–	–	0.25	0.18	–	–	0.11	0.11
**Eigenvector**	–	–	–	0.25	–	–	–	0.18

### Reach for selected promoters and FISH trial peer leaders

**Fig 3:**
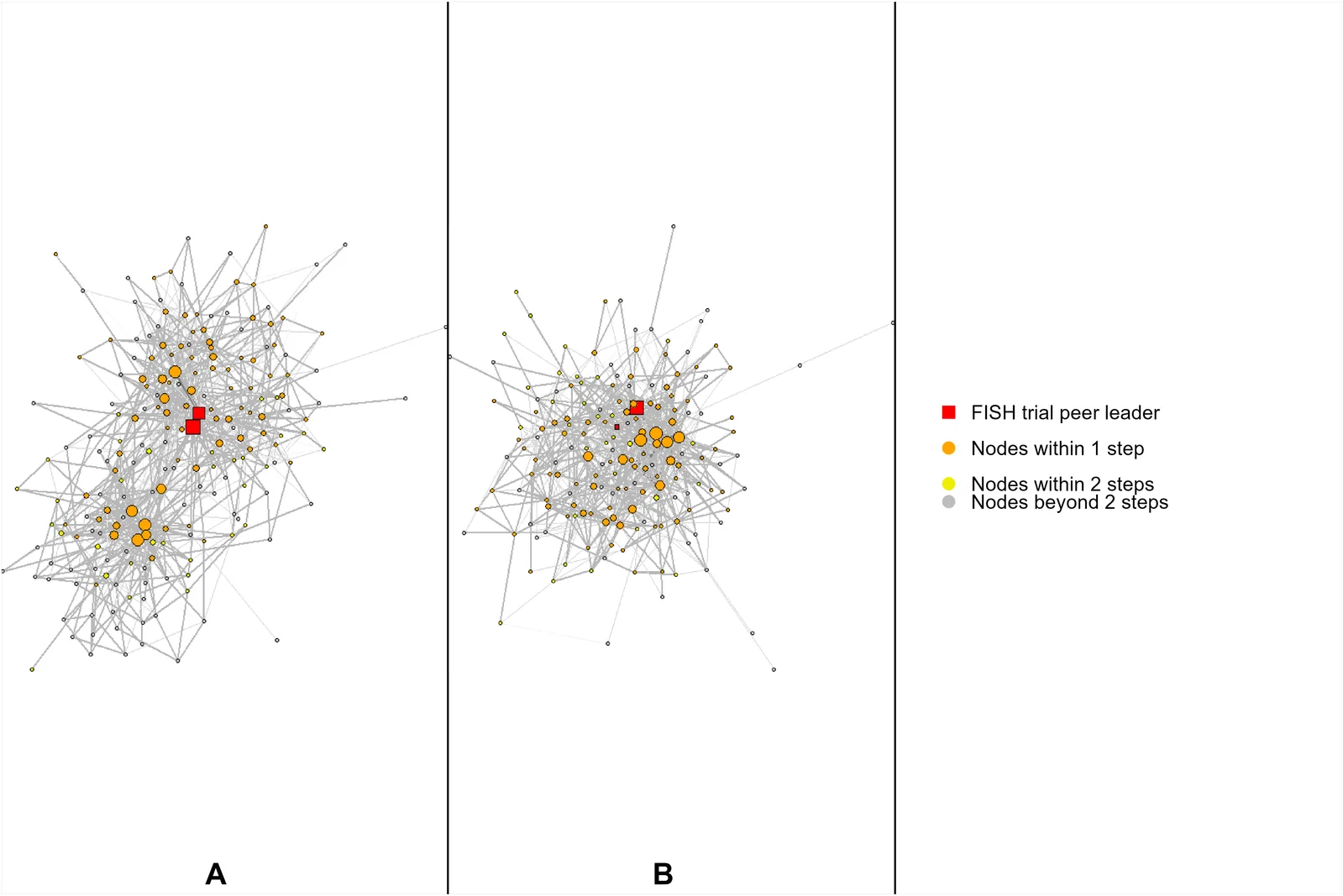
Network maps showing the reach of peer leaders within geographically distinct study clusters in Mangochi, Malawi. Each circle represents a fisherman (node). Line thickness is proportional to the domains of support provided by the tie.

We calculated the shortest path distances from each peer leader to all other nodes and identified those within the equivalent of two steps. In F001, 117 (58%) non-promoters were reachable within two steps of the FISH trial peer leaders (Fig 3A), whereas in F024, 125 nodes (73%) fell within this range (Fig 3B).

**Fig 4:**
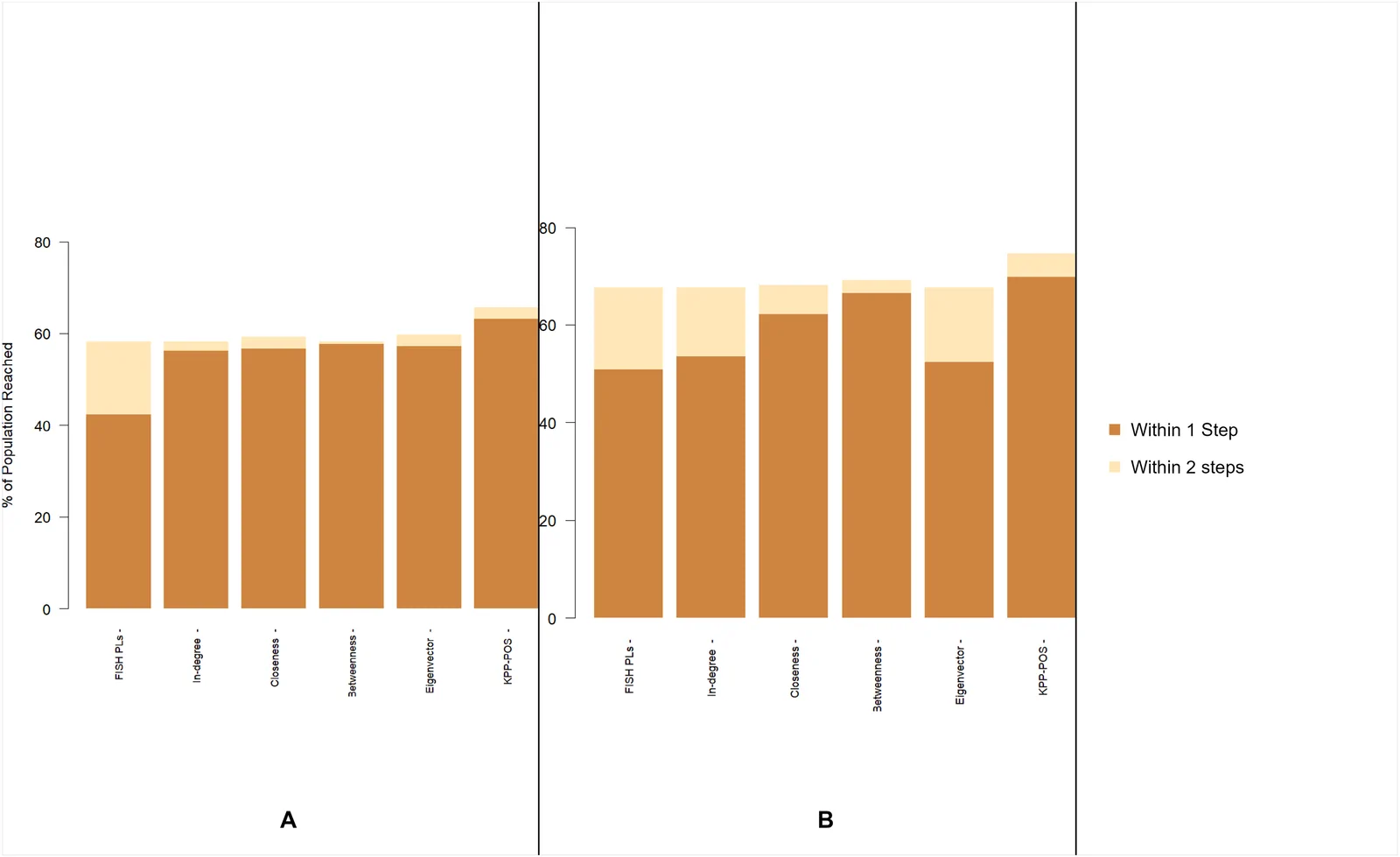
Reach of promoter sets classified by cluster, showing the percentage of individuals reached within one and two steps for each sociocentric method.

The sociocentric promoter sets selected in F001 and F024 reached most of the non-promoters within one step, with a few more reached within two steps (Figs 4A and 4B). In F001, the in-degree and betweenness sociocentric promoter sets showed equivalent reach to the FISH peer leaders, while in F024, both the in-degree and eigenvector sets matched the reach of the FISH peer leaders, despite the sociocentric promoter sets being larger. However, despite having similar reach, a greater proportion of the participants were reachable within one step of the sociocentric promoter sets compared to the FISH peer leaders. Other promoter sets demonstrated greater reach than the FISH peer leaders, with KPP-POS achieving the highest reach within two steps, 66% (F001) and 75% (F024).

## Discussion

This study found that the use of network information to inform promoter selection has promise in improving the diversity and reach of promoters and potentially enhancing intervention diffusion. This approach is critical for effective public health programming in diverse populations like fishing communities, where unique socio-cultural and occupational challenges shape health behaviors. This study contributes towards the practical approaches for using network analysis in optimizing peer-led health interventions in complex social settings. SNA has increasingly been used in LMICs to understand how naturally occurring social ties influence health behaviors and outcomes and to inform the design and delivery of public health interventions [29,31,37,45].

Our results on the network structure indicate that F001 had two dense cores linked by overlapping edges while F024 had a single large core with small periphery. F001’s structure could enable rapid message reinforcement between cores with risk of poor periphery penetration while F024’s structure could support uniform spread via core-periphery dynamics, though with fewer distinct entry points than multicore structures [46]. We also found that the promoters selected using sociocentric methods occupied diverse positions within the network, and their demographic profiles varied according to the selection method. The KPP-POS approach yielded the greatest diversity, reflecting its design to identify important nodes in different sub-sections of the network and ensure varied representation and maximum coverage [22,47,48]. By selecting dispersed, non-redundant sets, this could potentially aid practical rollout in dynamic settings by avoiding reliance on a few central figures. These findings align with recommendations that opinion leaders should represent a diversity of social groups to facilitate diffusion [21].

Additionally, more than half of the population in both clusters was reachable within two steps, and of these, over 80% were within one step of the identified promoters for all methods. This close proximity has significant potential since the likelihood of exposure to an intervention increases the more proximal the nodes are to a promoter and typically tapers off beyond three steps [49,50]. Consistent with this, studies of network-based HIV delivery among fishermen have successfully leveraged close social ties and demonstrated an increase in testing and linkage to care [29,51]. However, nodes selected based on sociocentric centrality measures did not demonstrate greater reach than the FISH trial peer leaders selected by peer nomination, except when using KPP-POS. This may be explained by two factors: first, the FISH trial peer nominations likely served as a proxy for individuals with high in-degree centrality; second, given the known correlations between centrality measures, there was substantial overlap among the top-ranked nodes selected by each centrality measure [3]. Thus, the neighborhoods of the promoter sets were similar to the FISH trial peer leaders.

This study used network level data to propose hypothetical sets of promoters that could potentially be used to deliver interventions similar to those in FISH trial in a future study. The limited use of network data in promoter selection could explain the modest impact on outcomes observed in most peer-led health interventions [30]. For example, in this study, we found that in cluster F024 — which performed poorly in the FISH trial — one of the peer leaders was consistently not selected by any of the sociocentric measures. Potentially, this peer leader may have contributed to the poor outcomes observed in this cluster, suggesting that who you choose to promote an intervention matters and their position within the network structure can influence intervention effectiveness. Using network analysis creates a systematic approach for promoter identification, but it has its challenges. These challenges include logistical barriers to network data collection, ethical concerns regarding data privacy, and measurement difficulties arising from the dynamic nature and fluid boundaries of social networks [23,52,53]. Moreover, accurately capturing social ties can be complicated by participants’ recall bias or reluctance to disclose sensitive relationships, which may affect the validity of the network data [52]. Despite these challenges, the potential of social network analysis to enhance health interventions remains promising [25,54].

When network data are available, it is efficient to select a method that maximizes reach within one or two steps and ensures a diverse, representative promoter set. However, a single method may not fully address all factors necessary to enhance intervention effectiveness. It has been shown that using multiple sociocentric measures for selecting promoters captures diverse aspects of individuals’ roles within the network that single measures may miss. As demonstrated by Huang et al., combining measures like degree, betweenness and eigenvector centrality allows for a more comprehensive evaluation of node importance and role, identifying both cores and bridges essential for effective information dissemination [55]. This approach enhances promoter selection by reflecting network positions that influence intervention reach and success beyond mere connectivity [55]. However, beyond the network-level factors it is important to consider and collect experiential factors and personality factors of the promoters as these also play a role in intervention diffusion [33].

There are some limitations in this study that must be acknowledged. Firstly, the study covered only two fisherman’s landing sites and only included fishermen, excluding other important social actors to fishermen, e.g., women in the fishing trade and non-fishermen. Therefore, we may have excluded actors that potentially play an important role in information diffusion, and we could not explore assortative mixing by characteristics such as gender or occupation. Secondly, we are likely not to have captured the whole network through our one-time measurement, as fishermen are highly mobile. Thus, similar to other sociocentric network studies, our data may have been affected by incomplete network bias [56–58]. Third, the reliability of the network data may be limited due to recall bias; although self-perceived networks are likely to be the most relevant ones for network interventions. Fourth, while we cannot draw strong conclusions about the promoter selection that maximizes reach, the data are interesting and may point to KPP-POS promoter approach as an important approach. Lastly, we were not able to use the selected promoter sets to explore their impact on intervention adoption. Future work could implement these selection methods and observe actual intervention uptake in these communities. Despite these limitations, this study possesses several strengths. The study provides rich and detailed data on the extent and structure of the social networks of fishermen in an understudied, underserved population. We were able to demonstrate that it is feasible to collect social network data in this population and setting supporting the potential for future research and targeted interventions. Furthermore, the use of multiple network measures provides a systematic and reproducible approach for promoter identification. These strengths ensure that the findings offer valuable and actionable insights into network-based intervention strategies for similarly underserved populations.

## Conclusions

This study demonstrates that network analysis, particularly the KPP-POS approach, holds promise for optimizing promoter selection in peer-led health interventions by enhancing diversity and reach. Our findings highlight that strategically identified promoters can achieve good reach within social networks, crucial for effective public health programming in complex settings like fishing communities. There is a need to consider both network metrics and qualitative factors for comprehensive promoter identification. Future research should implement these theoretical selections to validate their real-world impact on intervention diffusion.

## Supporting information

S1 Checklist Inclusivity in global researchf. (DOCX)

S1 Fig Network promoters selected by sociocentric measures within two geographically distinct study clusters (F001 and F024) in Mangochi, Malawi. First panel A and B show indegree promoters for F001 and F024, respectively; Second panel A and B show closeness promoters; Third panel A and B show betweenness promoters; Fourth panel A and B show eigenvector promoters; and Fifth panel A and B show KPP-POS promoters across the respective clusters. Each circle represents a fisherman (node), with circle size scaled proportionally to the respective sociocentric measure. Line thickness indicates the strength or number of domains of support provided by each tie.
(DOCX)

S2 Fig Selected promoters and their rankings across sociocentric measures in cluster F001. (DOCX)

S3 Fig Selected promoters and their rankings across sociocentric measures in cluster F024. (DOCX)

S1 Table Promoter characteristics. (DOCX)
