# Supplementary material for: Optimizing peer-led health interventions: A social network analysis approach for identifying influential fishermen in Malawi
Source: PLOS Glob Public Health. 2026 Sep 9;6(9):e0007185. doi: 10.1371/journal.pgph.0007185 (PMC13557381; doi:10.1371/journal.pgph.0007185)
Supplement: Supplementary file 2 [file pgph.0007185.s002.docx]

**S1 Fig**. Network promoters selected by sociocentric measures within two geographically distinct study clusters (F001 and F024) in Mangochi, Malawi. First panel A and B show indegree promoters for F001 and F024, respectively; Second panel A and B show closeness promoters; Third panel A and B show betweenness promoters; Fourth panel A and B show eigenvector promoters; and Fifth panel A and B show KPP-POS promoters across the respective clusters. Each circle represents a fisherman (node), with circle size scaled proportionally to the respective sociocentric measure. Line thickness indicates the strength or number of domains of support provided by each tie.
