# Supplementary material for: Optimizing peer-led health interventions: A social network analysis approach for identifying influential fishermen in Malawi
Source: PLOS Glob Public Health. 2026 Sep 9;6(9):e0007185. doi: 10.1371/journal.pgph.0007185 (PMC13557381; doi:10.1371/journal.pgph.0007185)
Supplement: Supplementary file 5 [file pgph.0007185.s005.docx]

S1 Table: Promoter characteristics

| Demographics | In-degree | | Closeness | | Betweenness | | Eigenvector | | KPP-POS | | **General popn** | |
| --- | --- | --- | --- | --- | --- | --- | --- | --- | --- | --- | --- | --- |
|  | F024 | F001 | F024 | F001 | F024 | F001 | F024 | F001 | F024 | F001 | **F024** | **F001** |
| Mean age (SD) | 41.3  (9.1) | 44.7  (8.6) | 37.7 (10.7) | 48.6 (10.1) | 38.5 (9.6) | 43.0  (11.2) | 41.7 (10.7) | 42.9  (9.6) | 29 (10.5) | 34.9 (12.7) | **31.7**  **(10.0)** | **37.4 (14.7)** |
| Highest level of education (n [%]) | | | | | | | | | | |  |  |
| No education | - | 2 (20) | - | 1 (10) | 1 (10) | 1 (10) | - | 1 (10) | 2 (20) | 1(10) | **9 (5)** | **10 (5)** |
| Primary | 5 (50) | 6 (60) | 7 (70) | 5 (50) | 8 (80) | 7 (70) | 2 (20) | 5 (50) | 7 (70) | 4(40) | **110(65)** | **86(43)** |
| Secondary | 5 (50) | 2 (20) | 3 (30) | 3 (30) | 1 (10) | 2 (20) | 8 (80) | 4 (40) | 1 (10) | 5(50) | **49 (29)** | **101(50)** |
| Tertiary | - | - | - | 1 (10) |  | - | - | - | - | - | **1 (1)** | **4 (2)** |
| Able to read one page? (n [%]) | | | | | | | | | |  |  |  |
| Yes | 10(100) | 6 (60) | 10(100) | 8 (80) | 8 (80) | 8 (80) | 10(100) | 8 (80) | 8 (80) | 9 (90) | **183(91)** | **144(85)** |
| Ever tested for HIV? (n [%]) | | | | | | | | | | |  |  |
| Yes | 10(100) | 10(100) | 9 (90) | 10(100) | 10(100) | 10(100) | 10(100) | 10(100) | 10(100) | 9(90) | **191(95)** | **146(86)** |
