# Supplementary material for: Optimizing peer-led health interventions: A social network analysis approach for identifying influential fishermen in Malawi
Source: PLOS Glob Public Health. 2026 Sep 9;6(9):e0007185. doi: 10.1371/journal.pgph.0007185 (PMC13557381; doi:10.1371/journal.pgph.0007185)
Supplement: Supplementary file 7 [file pgph.0007185.s004.docx]

**S3 Fig:** Selected promoters and their rankings across sociocentric measures in cluster F024
